# The Role of CEUS in the Diagnosis and Follow-Up of Pleuropulmonary Diseases and Interventional Procedures

**DOI:** 10.3390/jcm15062292

**Published:** 2026-03-17

**Authors:** Andrea Boccatonda, Alice Brighenti, Daniel Piamonti, Giulia Bandini, Giulia Fiorini, Luigi Vetrugno, Giampietro Marchetti, Esterita Accogli, Carla Serra, Damiano D’Ardes

**Affiliations:** 1 Diagnostic and Therapeutic Interventional Ultrasound Unit, IRCCS Azienda Ospedaliero-Universitaria di Bologna, Policlinico Sant’Orsola-Malpighi, Via Massarenti n 9, 40138 Bologna, Italy; alice.brighenti3@studio.unibo.it (A.B.); carla.serra@aosp.bo.it (C.S.); 2 Department of Public Health and Infectious Diseases, Sapienza University of Rome, 00185 Rome, Italy; daniel.piamonti@uniroma1.it; 3 Pulmonary Critical Care Unit, Policlinico Umberto I Hospital, 00185 Rome, Italy; 4 Department of Internal Medicine, University Hospital Careggi, 50134 Florence, Italy; giulia.bandini@unifi.it; 5 Cardiovascular Medicine Unit, IRCCS Azienda Ospedaliero-Universitaria di Bologna, 40138 Bologna, Italy; giulia.fiorini@aosp.bo.it; 6 Emergency Department, Anesthesia and Intensive Care, Health Integrated Agency of Friuli Centrale, 33028 Tolmezzo, Italy; luigi.vetrugno@asufc.sanita.fvg.it; 7 Pulmonology Unit, ASST Spedali Civili, 25123 Brescia, Italy; marchetti.giampietro@libero.it; 8 Department of Internal Medicine, Centre of Research and Learning in Ultrasound, Maggiore Hospital, 40138 Bologna, Italy; esterita.accogli@ausl.bologna.it; 9 Institute of “Clinica Medica”, Department of Medicine and Aging Science, Gabriele d’Annunzio University of Chieti and Pescara, 66100 Chieti, Italy; damiano.dardes@unich.it

**Keywords:** CEUS, lung ultrasound, pneumonia, pulmonary embolism, COVID-19, lung cancer

## Abstract

**Background:** Contrast-enhanced ultrasound (CEUS) recently emerged as a valuable imaging modality for evaluating pleuropulmonary diseases. By combining morphological information from conventional B-mode ultrasound with real-time assessment of microvascular perfusion, CEUS can provide functional insights that improve diagnostic accuracy, guide interventions, and support patient surveillance. **Methods:** This review summarizes the current evidence on the use of CEUS in major pleuropulmonary disorders, including pneumonia, pleural effusion, pulmonary embolism, neoplasms, and COVID-19-related lung injury. The most relevant clinical studies and meta-analyses were analyzed, focusing on CEUS parameters, diagnostic performance, and integration with other imaging techniques. **Results:** CEUS enables the differentiation between inflammatory, ischemic, and malignant lesions through qualitative and quantitative analyses of enhancement patterns. Early and homogeneous enhancement is typical of inflammatory or infectious processes, whereas heterogeneous or delayed enhancement with early washout strongly suggests malignancy or ischemia. In pneumonia and pleural infections, CEUS identifies non-perfused or necrotic areas, guiding drainage and evaluating therapeutic responses. In pulmonary embolism, it reveals avascular consolidations corresponding to infarction, even when CT angiography is inconclusive. For peripheral lung tumors, CEUS assesses angiogenesis and vascular supply, correlating perfusion parameters with histopathology, and improving biopsy targeting. Furthermore, in COVID-19 pneumonia, CEUS can detect microvascular alterations related to thrombosis and fibrosis. **Conclusions**: CEUS is a safe, noninvasive, and radiation-free technique that provides unique real-time information on pulmonary perfusion. Its integration with conventional ultrasound enhances diagnostic precision, optimizes interventional guidance, and allows for dynamic monitoring of treatment response. Future developments in quantitative analysis, artificial intelligence, and targeted contrast agents are expected to further expand CEUS clinical applications in pleuropulmonary imaging.

## 1. Introduction

In recent years, lung ultrasound has gained increasing importance in the assessment of pleuropulmonary diseases and has become an integral part of chest imaging. This development has been driven by technological advances, the availability of high-performance portable equipment, and the clinical need for rapid, safe, and repeatable diagnostic methods, a need that became particularly evident during the COVID-19 pandemic [1]. In B-mode imaging, ultrasound allows for the evaluation of the pleural line, pleural space, and subpleural lung structures, enabling the identification of effusion, thickening, and consolidation. However, the presence of alveolar air induces a marked acoustic impedance mismatch, limiting the penetration of the ultrasound beam into the normally aerated lung parenchyma. Consequently, this technique is primarily useful for assessing pleural and subpleural lesions. Consolidations appear as hypoechoic or heterogeneous areas contiguous with the pleura, sometimes containing air or fluid bronchograms. Despite its high sensitivity, ultrasound morphology alone does not always allow for a reliable distinction between inflammatory, infectious, or neoplastic processes [2,3]. To overcome these limitations, CEUS has been introduced, allowing dynamic real-time assessment of microvascular perfusion. CEUS uses gas microbubbles stabilized by a phospholipid shell, such as sulfur hexafluoride (SF_6_) contained in Sonovue^®^ (Bracco Imaging S.p.A., Milan, Italy). In adults, the standard administered dose is 2.4 mL, injected at a rate of 1–2 mL/s, followed by a 10 mL saline solution (0.9% NaCl) bolus. Once injected intravenously, these microbubbles reflect low-intensity ultrasound waves and enhance blood flow visualization within tissues or lesions. The basic principle of CEUS is the acoustic resonance of microbubbles, which oscillate when struck by ultrasound waves, thereby amplifying the reflected signal compared to the surrounding tissues and allowing for a dynamic representation of tissue perfusion [1,3]. “Contrast-specific” imaging modes with a low mechanical index (MI < 0.1) allow visualization of the passage and distribution of the contrast agent within lesions visible in B-mode, providing functional information that complements morphological findings [2,3]. CEUS is increasingly being investigated in lung diseases and other clinical settings, including pediatric neurocritical care, where it may enable real-time bedside assessment of cerebral perfusion and microvascular circulation in critically ill children [4,5].

The aim of this review is to provide a comprehensive overview of the current evidence on the role of CEUS in pleuropulmonary diseases, highlighting its diagnostic value in the characterization of peripheral lung lesions, its contribution to the differential diagnosis of consolidative syndromes, and its emerging role in interventional guidance and clinical monitoring.

## 2. Literature Search Strategy

This narrative review was conducted through a structured literature search aimed at identifying the most relevant studies on the clinical applications of CEUS in pleuropulmonary diseases. Electronic databases, including PubMed/MEDLINE, Scopus, and Web of Science, were searched for articles published up to January 2026. The search strategy included combinations of the following keywords: “contrast-enhanced ultrasound”, “CEUS”, “lung ultrasound”, “pleuropulmonary lesions”, “pneumonia”, “pulmonary embolism”, “lung cancer”, “pleural disease”, and “thoracic ultrasound”. Original research articles, systematic reviews, and meta-analyses investigating the diagnostic role of CEUS in pleuropulmonary diseases were considered eligible. Additional relevant publications were identified through manual screening of reference lists of selected articles and recent guideline documents from international ultrasound societies.

## 3. Choice of Probe and Acoustic Window

The selection of the transducer is crucial for optimizing the image quality. Low-frequency convex probes (2–5 MHz) are the standard choice for overall thoracic examination, as they provide good penetration and adequate sensitivity to microbubbles. In contrast, high-frequency linear probes (7–12 MHz) are preferred for evaluating superficial structures, such as the pleural line and chest wall. According to WFUMB and EFSUMB guidelines, it is essential to select dedicated CEUS presets with a mechanical index (MI) < 0.1, disable noise reduction filters, and record the entire arterial and parenchymal sequence [2,3]. The acoustic window should be selected based on the lesion location and patient position. The posterior or lower lateral intercostal windows between the scapular and posterior axillary lines generally provide the best visualization of subpleural lesions and effusions. In uncooperative patients or those in the supine position, the anterior parasternal or subcostal windows can also be used. Image quality also depends on the angulation of the ultrasound beam relative to the pleura and the respiratory stability. These parameters should be optimized to minimize artifacts and ensure adequate reproducibility of the examination [1,2,3].

## 4. Vascular Physiology and Contrast Phases

The lung parenchyma has a dual arterial blood supply consisting of the pulmonary and bronchial arteries. The former, low-pressure vessels originate from the right ventricle and are involved in gas exchange. The systemic vessels arise from the aorta, supply the airways and interstitial tissue, and are characterized by a high angiogenic capacity. The ratio between pulmonary and bronchial blood flow varies according to the underlying disease etiology and influences the perfusion pattern observed with CEUS [1,3]. This dual vascular supply gives rise to two main contrast phases: an arterial phase (15–40 s after injection), corresponding to the arrival and peak of contrast in the pulmonary circulation, and a parenchymal phase (beyond 40 s), which reflects the contribution of the bronchial and systemic circulation. Qualitative analysis of the enhancement pattern allows differentiation between various etiologies. Early and homogeneous enhancement is typical of inflammatory or infectious lesions, whereas heterogeneous or delayed enhancement, often associated with early washout, suggests a neoplastic or ischemic origin [1]. For the accurate execution and interpretation of pulmonary CEUS, it is essential to precisely assess 3 key parameters: enhancement time, enhancement intensity, and washout time. The arrival time (AT) can help to determine the vascular origin of the lesion. An enhancement AT < 6 s indicates supply from the pulmonary arteries, whereas an AT > 6 s suggests systemic bronchial vascularization. Comparing the enhancement of the lesion with that of the aerated lung and chest wall also aids in identifying the source of blood supply. The enhancement intensity (marked, moderate, or absent) reflects the degree of perfusion. Heterogeneous enhancement is more frequently associated with malignant lesions, although it has moderate sensitivity and specificity. The washout time (TW), defined as the disappearance of contrast from the lesion, is a crucial indicator of malignancy. Early washout (<60 s) has high specificity (100%) but low sensitivity for malignant lesions, whereas delayed or absent washout is more typical of benign processes [1,6]. Therefore, the combined assessment of AT, intensity, and TW provides crucial information on the vascular nature and potential malignancy of pulmonary lesions on CEUS [1,3].

## 5. Safety and Risk–Benefit Ratio

CEUS is a safe technique with an extremely low rate of adverse reactions (<1:10,000), which are generally mild and transient [7]. However, in patients with significant cardiopulmonary disease or severe pulmonary hypertension, a transient increase in microvascular load may pose a potential risk, and contrast arrival time may be prolonged. According to the product information of sulfur hexafluoride microbubbles (SonoVue^®^, Bracco Imaging), CEUS is contraindicated in patients with known hypersensitivity to the contrast agent, right-to-left cardiac shunts, severe pulmonary hypertension defined as pulmonary artery pressure >90 mmHg, uncontrolled systemic hypertension, and adult respiratory distress syndrome. In addition, caution is recommended in patients with unstable cardiovascular conditions, including recent acute coronary syndrome, angina at rest within the previous 7 days, acute heart failure, NYHA class III–IV heart failure, or severe arrhythmias. Therefore, the EFSUMB and WFUMB guidelines recommend an individual assessment of the risk–benefit ratio, particularly in patients with hemodynamic or ventilatory impairment, intracardiac shunts, or severe cardiac failure. In such cases, the examination should be performed in a monitored setting with immediate availability of medical assistance [2,3]. Clinical monitoring is recommended during and for at least 30 min after administration. It should also be acknowledged that many of the contraindications reported in the product information are largely precautionary and are not supported by a large body of real-world clinical data specifically addressing thoracic CEUS applications. As a result, the actual clinical relevance of some restrictions remains a matter of ongoing discussion [8]. Nevertheless, the available evidence consistently indicates that CEUS is associated with an excellent safety profile when used according to recommended clinical practice [7,9,10].

## 6. Evaluation and Differential Diagnosis of Consolidative Syndromes

**Figure 1:**
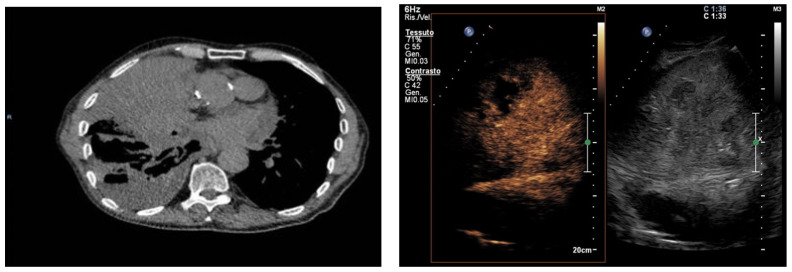
Multimodal imaging of extensive pulmonary consolidation with hepatization and central necrosis. Axial computed tomography (CT) image showing a large pulmonary consolidation with a hepatized appearance. Contrast-enhanced ultrasound (**left**) and corresponding B-mode ultrasound (**right**) demonstrating a solid-appearing consolidation with a non-enhancing intralesional area consistent with necrosis.

**Figure 2:**
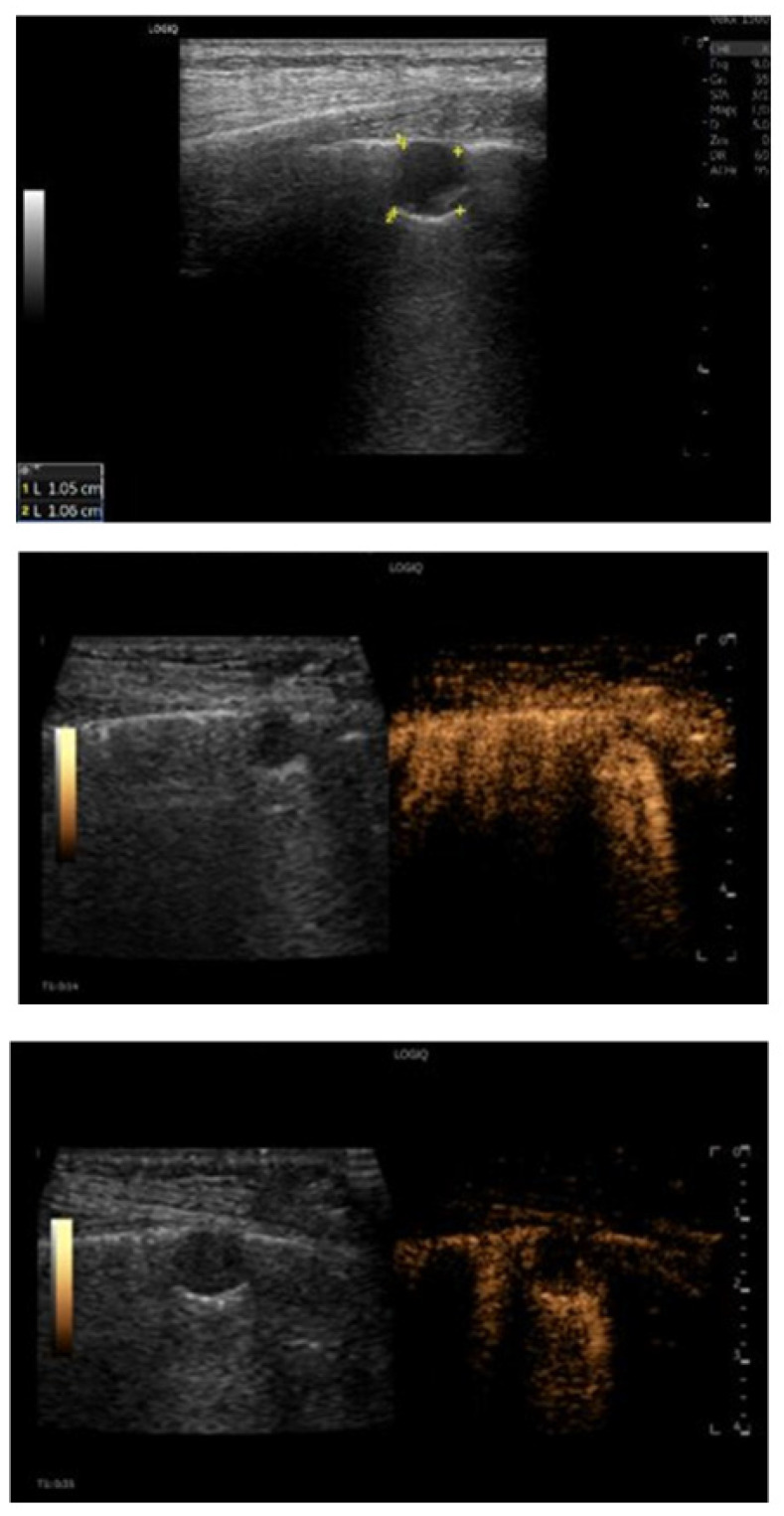
B-mode and contrast-enhanced ultrasound (CEUS) features of a peripheral pulmonary metastasis from breast carcinoma. Ultrasound demonstrates a small, oval-shaped peripheral pulmonary lesion showing early contrast enhancement followed by rapid and complete wash-out, a perfusion pattern consistent with metastatic disease. Final diagnosis was pulmonary metastasis from breast carcinoma.

The consolidative syndrome becomes visible on ultrasound because of the loss of alveolar ventilation and a simultaneous increase in the fluid component due to exudate, transudate, and/or inflammatory-necrotic infiltrates. The progressive disappearance of normal air content within the alveoli leads to the loss of typical pulmonary parenchymal artifacts, with consolidation becoming evident once it reaches and involves the pleural surface [1,11,12]. In B-mode imaging, subpleural consolidations appear as hypoechoic or heterogeneous areas with irregular margins and, occasionally, mobile air bronchograms. In infectious forms, such as pneumonia, the ultrasound pattern is generally more homogeneous, and the pleural line remains relatively regular. In contrast, in neoplastic processes, irregular margins, pleural discontinuity, and loss of local pleural motion are more frequently observed [1,2]. CEUS plays an increasingly important role in the evaluation of peripheral pleuropulmonary lesions, allowing the integration of B-mode morphological data with dynamic microvascular information (Figure 1). In this context, CEUS adds the ability to analyze microvascular perfusion and qualitatively and quantitatively assess contrast distribution. In general, inflammatory lesions typically show early and homogeneous enhancement that persists over time, whereas malignant lesions are characterized by heterogeneous, delayed enhancement with rapid washout, reflecting neoangiogenesis and the predominance of bronchial arterial flow [1,13] (Figure 2) (Table 1).

Several studies have aimed to describe the role of CEUS in the differential diagnosis of benign and malignant pleural lesions (Table 2). Tang et al. evaluated the Time Difference of Arrival (TDOA) in 96 patients with histologically confirmed peripheral pulmonary lesions to differentiate benign from malignant ones. The TDOA, defined as the difference in contrast arrival time between the lesion and adjacent lung tissue, was significantly higher in malignant lesions than in benign lesions (4.03 s vs. 1.09 s; *p* < 0.001). With a cutoff of 2.42 s, it achieved high diagnostic accuracy (AUC 0.894, sensitivity 86.3%, specificity 88.9%). The authors concluded that TDOA is a simple and reliable CEUS parameter, less affected by physiological variables, and useful for distinguishing malignant from benign lesions and guiding biopsy selection [13]. In 2021, a multicenter study was conducted on 812 patients to develop and validate a predictive ultrasound model capable of distinguishing benign from malignant subpleural lung lesions (SPLs) by integrating B-mode and CEUS (SonoVue^®^) parameters. Among the 18 variables analyzed, six were included in the final model: angle between the lesion and chest wall, baseline intensity, difference and ratio of contrast arrival time (AT), vascular sign, and type of non-enhancing area. The model demonstrated high diagnostic accuracy (C-statistic 0.974 in the development cohort and 0.980 in the validation cohort), with sensitivity ranging from 92.9% to 94.8% and specificity from 92.4% to 92.6%, outperforming the conventional criteria. The authors concluded that the multiparametric combination of CEUS and B-mode indices enables accurate and reproducible assessment of malignancy probability, improving the differential diagnosis of SPLs [14]. Zadeh et al., in a retrospective study conducted on 83 patients with pleural effusion of unknown origin, evaluated the role of CEUS (SonoVue^®^) in distinguishing between malignant and benign pleural effusions, in combination with B-mode ultrasound and cytology. The results showed that pleural thickening with marked enhancement and pulmonary consolidation with heterogeneous enhancement were significantly more frequent in malignant effusions. The use of CEUS increased the diagnostic sensitivity from 69.2% to 92.3% and specificity from 63.0% to 90.0%, improving the overall accuracy from 66.7% to 87.5% compared with B-mode ultrasound alone. The authors concluded that CEUS represents a valuable complement to cytology in the evaluation of pleural effusions of unknown cause, and that a CEUS pattern characterized by markedly enhanced pleural thickening and heterogeneous consolidation may indicate a malignant etiology [15]. Findeisen et al. analyzed 63 patients with pleural wall lesions with confirmed histological or cytological diagnosis to assess the effectiveness of CEUS (SonoVue^®^) in differentiating between benign and malignant forms. Malignant lesions (61.9%) were significantly thicker than benign lesions (31.2 ± 18.9 mm vs. 18.3 ± 15.1 mm; *p* = 0.003), with a 15 mm cut-off showing 78.6% sensitivity and 74.1% specificity. Among the CEUS parameters, marked contrast enhancement (EE) was much more frequent in malignant lesions (92.3% vs. 45.8%; *p* < 0.001), whereas contrast homogeneity (HE) showed no significant difference. The interobserver agreement for EE was good (κ = 0.73). The authors concluded that marked and heterogeneous enhancement is strongly suggestive of malignancy, although it may also occur in chronic or tuberculous pleuritis owing to reactive neoangiogenesis. Although CEUS does not replace CT or PET/CT, it is a valuable bedside functional tool for assessing perfusion, guiding biopsy, and monitoring the evolution of pleural lesions [16]. In a further prospective study conducted in 2022 on 50 patients with pleural thickening of unknown origin, the authors compared high-frequency B-mode ultrasound and CEUS (SonoVue^®^) for differential diagnosis. The final diagnosis was confirmed via pleural biopsy. Malignant lesions (*n* = 30) showed greater thickness, irregular or nodular morphology, and rapid but heterogeneous enhancement (“fast-in/fast-out”), whereas benign lesions (*n* = 20) exhibited uniform thickening with homogeneous and persistent enhancement (*p* < 0.05). Quantitative analysis of time–intensity curves (TIC) demonstrated shorter arrival time (AT) and time to peak (TTP), as well as higher peak intensity and area under the curve in malignant pleuritis. The diagnostic accuracy (AUC) was 0.819 for B-mode, 0.848 for CEUS, and 0.975 for the combination of morphological and perfusion parameters, indicating that the combined use of both techniques significantly improved the discriminative capability. In conclusion, high-frequency CEUS integrated with morphological ultrasound evaluation represents a noninvasive and highly accurate approach for the differential diagnosis of pleural diseases and targeted selection of biopsy sites [17]. In a prospective study involving 317 patients with peripheral pulmonary consolidations, the authors evaluated the diagnostic value of contrast arrival time (AT) and other dynamic CEUS (SonoVue^®^) parameters for differentiating between benign and malignant lesions. The mean AT did not differ significantly between the two groups (26.8 vs. 27.0 s; *p* = 0.39), and a 10-s cutoff showed low accuracy (47.6%) and sensitivity (5.3%). Similarly, a washout time (WOT) > 300 s did not improve discrimination (accuracy, 53.6% sensitivity 16.5%). In this study, no significant differences in enhancement patterns were observed, although squamous cell carcinomas exhibited a slightly delayed AT compared with other histotypes. The authors concluded that dynamic CEUS parameters alone do not allow reliable differentiation between inflammatory and neoplastic consolidations because of the considerable overlap of perfusion patterns. Chest CT remains the gold standard for characterization and staging, while CEUS retains a complementary role in the evaluation of peripheral lesions and in guiding biopsies [18]. In a recent systematic review and meta-analysis, the authors evaluated the diagnostic accuracy of CEUS (SonoVue^®^) in differentiating between benign and malignant subpleural lesions, including 10 studies with a total of 2622 patients. The pooled analysis demonstrated high diagnostic accuracy, with an overall sensitivity of 0.95 (95% CI: 0.93–0.97), specificity of 0.93 (95% CI: 0.90–0.95), and an SROC curve AUC of 0.97. Among the qualitative parameters analyzed, early washout emerged as the most specific marker of malignancy (specificity 0.98; 95% CI: 0.92–1.00), whereas heterogeneous enhancement showed moderate sensitivity (0.57) and limited specificity (0.51). Homogeneous and persistent enhancement was more frequently associated with benign lesions (sensitivity, 0.43; specificity, 0.49). The absence of enhancement or early contrast arrival did not demonstrate discriminative value. The authors concluded that heterogeneous enhancement combined with early washout represents the pattern most strongly associated with malignancy, indicating that CEUS has a high diagnostic accuracy for detecting malignant subpleural lesions. Furthermore, the integrated assessment of multiple CEUS findings, rather than single parameters alone, allows for excellent overall diagnostic performance, confirming CEUS as a high-yield functional technique for microvascular characterization of peripheral lung lesions [19]. A 2025 systematic review highlighted that combining CEUS parameters with real-time cytological evaluation (ROSE) significantly increased the diagnostic yield of biopsies (97.6% with CEUS vs. 84% with conventional ultrasound) without an increase in complications. Among the quantitative parameters analyzed, the time difference of arrival (TDOA) and delta arrival time (ΔAT) ≥ 2.5 s proved to be the best discriminators of malignancy, whereas isolated parameters such as arrival time or washout alone showed limited reliability. Multiparametric logistic regression models combining various CEUS variables (AT, TTP, peak intensity, and area under the curve) achieved near-excellent accuracy (C-statistic > 0.97), outperforming single-threshold approaches. Overall, the study confirms that quantitative CEUS, particularly when integrated with complementary cytological or ultrasound tools, is a reliable, reproducible, and highly accurate method for microvascular characterization and biopsy planning in subpleural lesions [20]. Cong et al., in a retrospective study of 212 patients with subpleural lung lesions (SPLs) who underwent CEUS (SonoVue^®^), analyzed the morphological and dynamic characteristics of necrotic areas to determine their value in the differential diagnosis between benign and malignant SPLs. Malignant lesions (*n* = 113) were more common in older and male patients, were larger, and more frequently exhibited necrotic areas with burr-like enhancing margins than benign lesions (*p* < 0.05). Additionally, the contrast arrival time (AT) was longer in malignant lesions than in benign lesions (11.1 vs. 9.5 s; *p* = 0.002). Multivariate analysis identified advanced age, male sex, lesion size, prolonged AT, and burr-like necrotic margins as independent predictors of malignancy. The authors concluded that CEUS evaluation of necrotic areas, particularly the presence of burr-like margins, can provide valuable additional information for the differential diagnosis of SPLs and for selecting the optimal biopsy target [21].

Overall, the available evidence suggests that CEUS may provide relevant functional information for the differential diagnosis of benign and malignant subpleural lung lesions. Among the various parameters investigated, heterogeneous enhancement and early washout consistently emerge as the most specific indicators of malignancy. Quantitative indices such as contrast AT and TDOA have also demonstrated diagnostic value in several studies, particularly when integrated into multiparametric models. However, the diagnostic performance of individual parameters may vary across studies, and considerable overlap between inflammatory and neoplastic lesions has been reported. Consequently, the combined assessment of multiple CEUS features—including enhancement pattern, washout kinetics, and vascular supply—appears to provide the highest diagnostic accuracy and should be interpreted in conjunction with morphological ultrasound findings and clinical context.

## 7. Pneumonia

**Figure 3:**
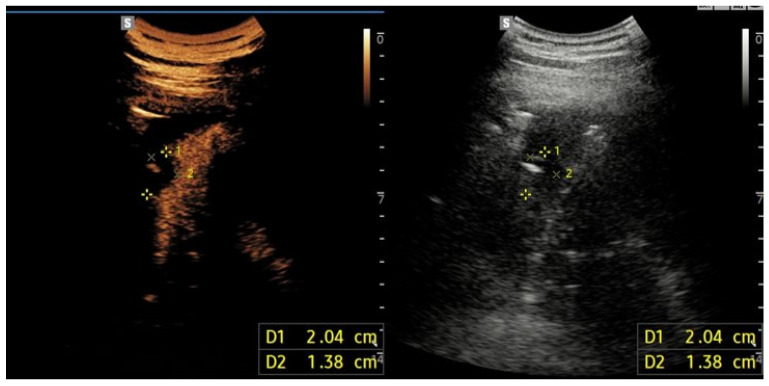
Contrast-enhanced ultrasound features of lobar pneumonia with intraparenchymal necrosis and associated pleural effusion. Contrast-enhanced ultrasound (**left**) and corresponding B-mode ultrasound (**right**) images show a lobar pulmonary consolidation with a well-defined, non-enhancing intralesional area consistent with necrosis (measured diameters indicated). A concomitant ipsilateral pleural effusion with complex, echogenic content is also observed.

On B-mode ultrasound, pneumonia may present with various morphological patterns, including subpleural consolidations that move with respiration, the presence of dynamic air bronchograms, and, in about half of the cases, an associated parapneumonic pleural effusion [1,22]. The so-called “lung hepatization” is a characteristic sign in which the parenchyma takes on a liver-like appearance due to the loss of normal alveolar air content and its replacement with inflammatory material. In such cases, ultrasound is particularly effective when the lesion is peripheral or in contact with the diaphragm. The identification of a hypoechoic bronchogram, distinguishable from vessels using color Doppler, and the observation of air movement within the bronchi are useful features for differentiating pneumonia from atelectasis [1,22,23]. Infectious pneumonia is one of the main consolidative lesions, and the use of CEUS allows for a more precise assessment of pulmonary perfusion and parenchymal viability, as demonstrated in previous studies. In a recent study, Giangregorio et al. evaluated the effectiveness of CEUS in diagnosing community-acquired pneumonia (CAP) in elderly patients and compared it with clinical assessment, chest radiography, and conventional ultrasound. Among 84 patients with suspected pneumonia, the final diagnosis confirmed 65 patients with CAPs and 19 with neoplasms. CEUS demonstrated a sensitivity of 96.9%, specificity of 100%, and overall diagnostic accuracy of 97.5% (AUROC 0.98), outperforming both radiography and standard ultrasound. CEUS enables precise and non-invasive characterization of peripheral lung lesions, effectively distinguishing between pneumonia and neoplasia, and may reduce the need for contrast-enhanced chest CT in elderly patients [24]. In the acute phase, pneumonia shows marked arterial vascularization, mainly through the pulmonary artery, with an initial rapid and homogeneous enhancement generally observed within 5–7 s after contrast injection, corresponding to the pulmonary arterial flow [1,25]. This perfusion pattern reflects the inflammatory nature and preservation of the arterial vascular bed, allowing pneumonia to be distinguished from other consolidative syndromes with different etiologies. In a 2012 study, Linde et al. retrospectively analyzed 50 patients with alveolar pneumonia to characterize the CEUS patterns and assess their clinical significance. They demonstrated that alveolar pneumonia predominantly (92% of cases) exhibits a type 1 CEUS pattern, characterized by pulmonary arterial vascularization and isoechoic and homogeneous enhancement, indicating preserved pulmonary arterial perfusion and representing the typical inflammatory perfusion pattern. In contrast, the rarer type 2 patterns, showing bronchial supply and heterogeneous or hypoechoic enhancement, have no prognostic value but may reflect vascular variants or more complex inflammatory states [25]. CEUS also allows differentiation between pneumonia and obstructive atelectasis, which, although showing rapid enhancement, presents a more compact, regular, and completely homogeneous pattern related to compensatory hyperperfusion but lacks vascular disorganization. In contrast, pneumonia displays a less homogeneous pattern with small hypoenhanced or non-enhanced areas, indicative of necrosis or microabscess formation [17]. On CEUS, uncomplicated pneumonia generally shows early and homogeneous enhancement with late washout (>60 s), consistent with pulmonary arterial perfusion. When the inflammatory process progresses to necrosis, the enhancement pattern changes owing to the recruitment of systemic circulation vessels, and the necrotic area appears non-enhancing (Figure 3). Pulmonary cavitations also show no enhancement, and in this context, hyperechoic air bubbles should not be mistaken for contrast signals [1].

Pulmonary abscesses form within pneumonic consolidations and recruit vessels from the systemic circulation, showing delayed peripheral enhancement with a non-perfused central area on CEUS, which is typical of a necrotic-purulent collection. In more advanced stages, CEUS allows the identification of necrotic–abscess complications by detecting central non-perfused areas surrounded by a hypervascularized rim (“rim enhancement”), which is characteristic of purulent or necrotic collections. However, this pattern can be mimicked by neoplasms with central necrosis or abscesses developing on pre-existing tumor lesions, making clinical correlation essential. Empyema, finally, is a purulent and often loculated pleural effusion that appears on ultrasound as a complex collection with septations, particulate material, and pleural thickening; on CEUS, it shows peripheral enhancement of the inflamed pleura, known as the “split pleura sign,” which helps differentiate it from non-infected or serous effusions [1,21]. In cases of complex parapneumonic effusion or pleural empyema, CEUS shows an absence of enhancement within septations and loculated cavities, making it useful for guiding drainage and assessing the presence of communication between pleural spaces. In selected cases, the intrapleural injection of a small, diluted dose of contrast agent can help visualize the cavities more clearly [15]. In some cases of acute lobar pneumonia, a delay in contrast uptake may be observed, which can be attributed to reactive vasoconstriction. However, the perfusion pattern remains consistent with that of the pulmonary arterial circulation, clearly differing from neoplastic lesions, which instead exhibit disorganized, low-resistance bronchial vascularization with delayed and heterogeneous enhancement [16]. During follow-up, CEUS allows monitoring of therapeutic response by documenting the progressive reduction in enhancement and non-perfused areas in regressing lesions, while the appearance or expansion of hypoperfused zones suggests progression toward necrosis or a poor response to antibiotic therapy [19]. CEUS can therefore provide additional information in monitoring disease progression and detecting multiloculated pleural collections, as well as in verifying drain patency and assessing the response to antibiotic therapy, thus offering a dynamic representation of perfusion and residual inflammatory activity [1]. In this context, Fu et al. evaluated the role of ultrasound and CEUS in the differential diagnosis of focal organizing pneumonia (FOP) and primary lung malignancy (PLM). A retrospective analysis was performed on 23 patients with FOP and 100 with PLM, comparing the ultrasound and CEUS parameters. Three factors were identified as predictors of FOP: presence of the bronchoaeric sign (OR = 6.18; *p* = 0.025), acute angle between the lesion margin and chest wall (OR = 7.12; *p* = 0.033), and homogeneous contrast enhancement (OR = 35.26; *p* = 0.01). The combination of these parameters achieved an area under the ROC curve (AUC) of 0.96, with a sensitivity of 95% and specificity of 82.6%, demonstrating a high diagnostic accuracy. In conclusion, integration with CEUS allows effective differentiation between FOP and PLM, as uniform pulmonary vascularization and homogeneous enhancement patterns are typical of FOP, whereas heterogeneity and bronchial arterial supply more often characterize malignant lesions [26].

Despite the characteristic enhancement patterns observed in infectious pneumonia, several diagnostic pitfalls must be considered in clinical practice. Certain non-infectious conditions, such as organizing pneumonia or granulomatous diseases, may present with CEUS findings that partially overlap with inflammatory consolidations, including relatively homogeneous enhancement and delayed washout. Similarly, necrotic tumors or metastatic lesions may occasionally mimic pulmonary abscesses when they show central non-enhancing areas surrounded by peripheral vascularized tissue. In these cases, the differentiation based solely on perfusion patterns may be challenging. Clinical history, laboratory findings, and complementary imaging modalities—particularly chest CT—remain essential for accurate interpretation. Therefore, CEUS should be considered a complementary functional tool rather than a stand-alone diagnostic modality in the evaluation of pulmonary consolidations.

## 8. COVID-19

During the COVID-19 pandemic, lung ultrasound emerged as a first-line diagnostic tool in the evaluation of patients with viral interstitial pneumonia, as recognized by the World Health Organization (WHO) guidelines, owing to its speed, portability, and lack of radiation exposure [1,27,28]. SARS-CoV-2 pneumonia is a form of interstitial pneumonia that typically shows multiple and confluent B-line artifacts, irregular thickening of the pleural line, and bilateral subpleural consolidations with a predominantly peripheral distribution on B-mode ultrasound [28]. Although these findings are not specific, they are highly suggestive of viral involvement and have been associated with greater clinical severity in patients presenting with extensive disease and multiple peripheral consolidations [27,29]. Ultrasound has also enabled bedside monitoring, particularly in intensive care units, reducing the risk of infection spread during patient transport and providing an alternative to chest CT in emergency settings [29,30,31,32]. A recent meta-analysis evaluated the effectiveness of lung ultrasound in detecting interstitial sequelae and fibrotic-like changes in patients with previous COVID-19 pneumonia and compared its performance with that of chest CT. Studies published up to July 2024 were included, with a total of 610 patients and a mean follow-up of three months. The analysis showed a high sensitivity of ultrasound (up to 98%) in identifying post-COVID alterations, while the specificity varied depending on the threshold used for detecting B-lines. The model with the higher threshold achieved the best overall diagnostic accuracy (AUC 0.93), confirming the ability of this technique to accurately detect residual fibrosis and parenchymal damage. Lung ultrasound therefore represents a reliable, sensitive, and non-invasive tool for the follow-up of post-COVID-19 patients, useful for monitoring pulmonary recovery and reducing the need for CT, although greater standardization of scanning protocols and B-line interpretation criteria is still required [33]. In this clinical context, CEUS has further expanded the diagnostic capabilities by providing information on pulmonary microvascular perfusion and parenchymal viability. In patients with COVID-19, CEUS can detect alterations in perfusion patterns within subpleural consolidations, highlighting hypo- or non-perfused areas suggestive of microthrombosis or peripheral pulmonary infarctions—phenomena well described in the pathogenesis of the disease [19]. Moreover, CEUS can help differentiate viral consolidations from overlapping bacterial consolidations. Secondary bacterial pneumonia tends to show areas of stronger enhancement and delayed washout, whereas purely viral lesions typically display weaker and often heterogeneous enhancement. In cases of suspected pulmonary infarction, CEUS also allows the identification of non-perfused areas within pre-existing consolidations, supporting an early diagnosis of ischemia or necrosis [1,21,34]. In a 2020 study, the use of CEUS was evaluated in patients with severe COVID-19 to analyze peripheral pleuropulmonary alterations. In 11 ventilated patients, CEUS revealed subpleural consolidations with irregular hyperemia and hypoperfused areas corresponding to microemboli or peripheral infarctions, which was consistent with the CT findings. Hypervascularized regions were indicative of active inflammation, whereas avascular areas suggested necrosis or ischemia. The authors concluded that CEUS is a useful bedside monitoring technique capable of identifying microvascular disturbances and thromboembolic phenomena in real time in severe cases of COVID-19 [35]. Overall, the integration of lung ultrasound and CEUS has played a crucial role in the management of COVID-19 pneumonia, enabling functional assessment of perfusion and non-invasive, repeatable monitoring of parenchymal damage. This combination now represents an important clinical tool both in the acute phase and during post-infectious follow-up, particularly in patients with subpleural involvement or thromboembolic complications.

## 9. Pulmonary Embolism and Pulmonary Infarction

Lung ultrasound is a useful tool in the diagnosis of pulmonary embolism, as it allows the identification of peripheral pulmonary infarctions, which are often responsible for pleuritic chest pain in affected patients [36,37]. When acute pain originates from a pulmonary condition, the lesion must reach the parietal pleura, as occurs in subpleural infarctions resulting from the occlusion of a pulmonary arterial branch by a thrombus [38]. Such occlusion causes a rapid collapse of the surfactant system with atelectasis and transudation of fluid into the parenchyma, leading to a local loss of alveolar air, thereby allowing ultrasound to visualize the typical wedge-shaped consolidation area of infarction [39]. On B-mode ultrasound, pulmonary infarction typically appears as a triangular or wedge-shaped area of consolidation, hypoechoic or anechoic, with a pleural base and the absence of an air bronchogram [1,34].

**Figure 4:**
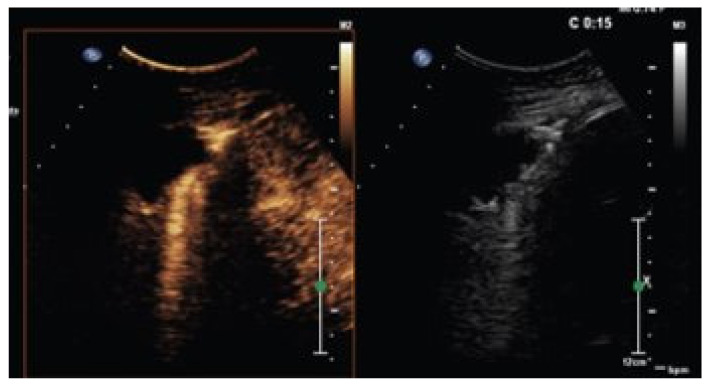
Contrast-enhanced ultrasound findings consistent with peripheral pulmonary infarction. Contrast-enhanced ultrasound (**left**) and corresponding B-mode ultrasound (**right**) images demonstrate a peripheral, wedge-shaped subpleural consolidation at the right lung base, characterized by absent contrast enhancement, consistent with pulmonary infarction.

CEUS allows for a more precise distinction of pulmonary infarction from other causes of pleuritic pain, as the infarct appears as an area lacking contrast enhancement in both the arterial and late phases, reflecting the absence of perfusion due to ischemic necrosis [1,40,41] (Figure 4). In some cases, however, a delayed peripheral enhancement may appear, caused by the perilesional inflammatory reaction or by bronchial reperfusion due to revascularization phenomena [1,42]. In a 2017 retrospective study, the authors analyzed 19 patients with suspected pulmonary embolism but negative CT findings for central emboli and assessed the role of B-mode ultrasound and CEUS in diagnosing peripheral embolic consolidations. All lesions were hypoechoic and predominantly triangular or wedge-shaped. On CEUS, 26% showed no enhancement, whereas the remaining 74% displayed a heterogeneous pattern indicative of hypoperfusion or pulmonary infarction. The authors concluded that CEUS can identify peripheral emboli not visible on CT, representing a useful tool in patients with a high clinical suspicion of pulmonary embolism and non-perfused pleural-based consolidations [43]. CEUS is also capable of detecting peripheral pulmonary infarctions in patients with a high clinical suspicion of pulmonary embolism but negative CT pulmonary angiography (CTPA) findings, supporting the complementary value of this technique in critical settings [1]. CEUS has also proven useful in the follow-up of patients with confirmed pulmonary embolism, allowing assessment of the slow and delayed regression of subpleural ischemic lesions—a feature that distinguishes them from inflammatory pneumonic processes [1,44]. In a 2022 study, Zadeh et al. retrospectively analyzed the role of B-mode thoracic ultrasound and CEUS in the follow-up of peripheral pulmonary lesions (PPLs) in patients with confirmed pulmonary embolism. In 27 patients, peripheral pulmonary lesions (≥5 mm) showed absent or heterogeneous enhancement on CEUS in 92.6% of cases, indicating hypoperfusion or ischemic necrosis. During short-term follow-up, approximately one-third of the patients exhibited changes in perfusion patterns, reflecting different stages of pulmonary infarction. At long-term follow-up, lesions were still visible in almost all cases but had decreased in size, demonstrating a slow yet progressive regression. This study therefore shows that combined monitoring with B-mode ultrasound and CEUS allows for better differentiation between ischemic and inflammatory lesions and supports the retrospective diagnosis of peripheral pulmonary infarction [44].

Although the absence of contrast enhancement within wedge-shaped subpleural consolidations is highly suggestive of pulmonary infarction, several conditions may produce similar CEUS appearances. Necrotic neoplasms, septic emboli, or cavitating infections may also present with non-perfused or poorly perfused areas, potentially mimicking ischemic lesions. Conversely, partial reperfusion through bronchial collateral circulation may lead to delayed or peripheral enhancement in true pulmonary infarctions, which can further complicate the interpretation. These potential overlaps highlight the importance of integrating CEUS findings with clinical probability scores, laboratory markers, and additional imaging such as CT pulmonary angiography when pulmonary embolism is suspected. In routine clinical practice, CEUS should therefore be interpreted within a multimodal diagnostic framework.

## 10. Pulmonary Neoplasms

**Figure 5:**
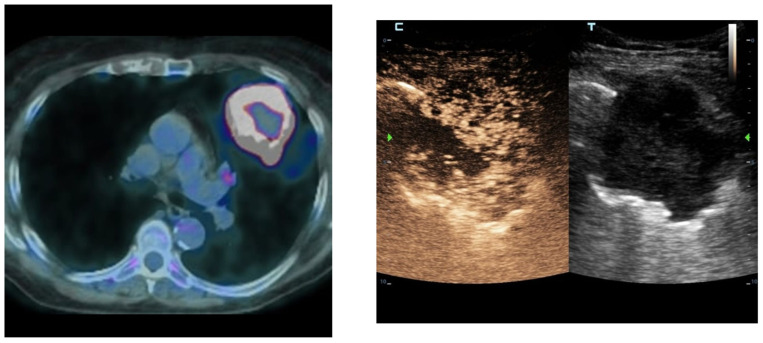
Multimodal imaging of a necrotic lung tumor. Left panel: axial fused positron emission tomography–computed tomography (PET/CT) image showing a hypermetabolic pulmonary mass with a central photopenic area consistent with intratumoral necrosis. Right panel: ultrasound imaging of the same lesion demonstrating a well-defined mass with a hypoechoic central necrotic component, shown on contrast-specific imaging (**left**) and corresponding B-mode ultrasound (**right**).

Concerning pulmonary neoplasms, only those that are peripheral or in contact with the pleura can be adequately assessed using lung ultrasound, which allows direct evaluation of the morphology and vascularization of the lesions [1]. In this context, CEUS represents an important technique to complement traditional imaging, offering advantages such as real-time dynamic imaging, a favorable cost–benefit ratio, and safety due to the absence of ionizing radiation [45]. CEUS enhances the assessment of tumor vascularization and angiogenesis, allowing differentiation between benign and malignant pulmonary nodules and enabling the monitoring of therapeutic responses (Figure 5).

It is also useful for lymph node evaluation, metastasis detection, and quantitative blood flow analysis. The main limitations of this technique include reduced effectiveness in deep lesions, individual variability, and operator dependency; however, advances in targeted contrast agents, multimodal imaging, and artificial intelligence are expected to further improve its reliability and clinical applications. From a technical standpoint, CEUS also enables quantitative blood flow analysis through time–intensity curves, correlating perfusion patterns with tumor aggressiveness and microvascular density [45]. In general, benign lesions tend to show earlier and more homogeneous enhancement, consistent with their pulmonary vascularization, whereas malignant neoplasms, which rely mainly on systemic bronchial circulation, usually present with heterogeneous or delayed enhancement, often associated with early washout. The latter, when combined with delayed enhancement, is highly suggestive of malignancy and warrants further histological investigation [1,6,46,47].

Several studies have explored the role of CEUS in this context.

Bai et al. studied 136 patients with subpleural lung lesions and demonstrated that CEUS effectively differentiates benign lesions (arborescent pattern) from malignant lesions (centripetal or eccentric pattern). Integration with color parametric imaging (CPI) significantly improved diagnostic accuracy (AUC from 0.68 to 0.86) and interobserver agreement, providing a more precise assessment of microperfusion and aiding in the distinction between small-cell lung cancer (SCLC) and non-small-cell lung cancer (NSCLC). In summary, the combination of CEUS and CPI represents a superior approach compared to CEUS alone for the characterization of subpleural lesions [48]. Li et al. evaluated 315 patients with primary peripheral lung carcinoma to explore the potential of CEUS for characterizing tumor vascularization. Lesions were classified based on enhancement time (TE): early enhancement indicated pulmonary vascularization (28.9%), whereas delayed enhancement was associated with bronchial vascularization (71.1%). Tumors with bronchial supply showed delayed, reduced, and heterogeneous enhancement with chaotic vascular distribution and greater necrosis, whereas those with pulmonary vascularization exhibited shorter TE, marked and homogeneous enhancement, and less necrosis. The study concluded that CEUS represents a promising and non-invasive tool for assessing microperfusion and the type of vascular supply in peripheral lung tumors, with potential implications for therapeutic planning and prognostic evaluation [49]. Safai Zadeh et al. included 54 patients with central lung carcinoma associated with obstructive atelectasis to determine the diagnostic value of CEUS combined with contrast-enhanced CT (CECT). The addition of CEUS increased the ability to distinguish tumor tissue from atelectatic parenchyma from 75.9% to 92.6%, significantly improving the diagnostic performance. Most central tumors showed a delayed bronchial perfusion pattern (89.6%) with reduced and homogeneous enhancement (91.7%) and rapid washout (<120 s, 79.2%), indicative of systemic blood supply and high neoplastic activity. CEUS also identified tumors that were not visible on CT alone or B-mode ultrasound, demonstrating high sensitivity in characterizing central lesions and distinguishing them from secondary atelectasis. The authors concluded that CEUS represents a useful, complementary, and non-invasive tool for improving diagnosis and therapeutic planning in this category of patients [50]. Some studies have analyzed how the histological type of the lesion influences the CEUS perfusion pattern. Findeisen et al. retrospectively analyzed 89 patients with peripheral lung carcinoma, comparing B-mode and CEUS findings to describe tumor vascularization in relation to histology. The study showed that bronchial arterial vascularization was predominant (72%) and was characterized by delayed enhancement (mean AT 17.6 s), hypoechoic and heterogeneous appearance, and frequent necrosis. Pulmonary arterial vascularization, observed in 28% of cases (mean AT 8 s), showed early enhancement, a hyperechoic and homogeneous appearance, and often the presence of an air bronchogram. From a histological standpoint, squamous cell carcinomas and small-cell carcinomas were almost exclusively supplied by bronchial arteries, whereas some adenocarcinomas, particularly the lepidic and papillary subtypes, displayed a pulmonary arterial supply. The authors suggested that tumors with low neoangiogenesis and noninvasive growth receive pulmonary arterial flow, whereas more aggressive and destructive tumors exhibit predominant bronchial vascularization. In conclusion, CEUS proves useful for assessing tumor microvascularization and distinguishing between different vascular patterns, providing information correlated with the biology and aggressiveness of peripheral lung carcinoma [47]. Du Yu Qing et al. evaluated the role of multimodal CEUS in the differential diagnosis of various types of peripheral lung carcinoma, analyzing a total of 102 patients with histologically confirmed peripheral lung cancer, including both small-cell lung carcinoma (SCLC) and non-small-cell lung carcinoma (NSCLC). The integration of CEUS, color parametric imaging (CPI), and time–intensity curve (TIC) analyses allowed for the detailed characterization of different perfusion patterns. The results showed that CPI clearly distinguished SCLC from NSCLC, as NSCLC lesions more frequently exhibited a centripetal enhancement pattern, whereas SCLC showed an eccentric pattern. In differentiating adenocarcinoma from squamous cell carcinoma, younger age, shorter contrast arrival time, prolonged washout time, and absence of internal necrosis were identified as predictors of adenocarcinoma. The resulting diagnostic model achieved high accuracy (AUC: 0.861). Multimodal CEUS proved to be a non-invasive and highly informative technique for the histological characterization of peripheral lung tumors, capable of accurately distinguishing between SCLC and NSCLC subtypes and providing functional information on tumor vascularization useful for therapeutic planning [51]. Despite these observations, there are many exceptions. For example, some less aggressive neoplasms may mimic inflammatory processes, and false positives may arise from chronic conditions such as granulomatous lesions or organizing pneumonias [1]. Therefore, pulmonary CEUS is a valuable tool for the microvascular evaluation of peripheral neoplasms; however, it should be interpreted with caution and always integrated with clinical and histological data.

Pulmonary metastases can only be detected by ultrasound when they reach the pleural surface or are in close contact with the costal or diaphragmatic pleura. When located peripherally, they can be identified using lung ultrasound, showing features similar to those of primary lung neoplasms. On CEUS, metastases tend to exhibit delayed, intense, and heterogeneous enhancement, often accompanied by early washout, generally within 60 s after contrast administration, which is considered one of the most indicative findings of malignancy [1,47]. Kroenig et al. evaluated the perfusion of peripheral pulmonary metastases using CEUS and correlated the findings with the vascular patterns identified by CD34 immunohistochemistry. In 54 patients, CEUS demonstrated a bronchial arterial enhancement pattern in the vast majority of cases (92.6%), whereas only a few showed pulmonary arterial supply. The lesions were characterized by marked heterogeneous enhancement with rapid washout, indicative of hypervascularization and bronchial-derived neoangiogenesis, which was later confirmed by CD34 analysis. Even in rare lesions showing a pulmonary pattern, a bronchial vascular component was present. These findings confirm that pulmonary metastases typically exhibit a CEUS pattern consistent with bronchial arterial supply and early washout, making CEUS a valuable tool for vascular assessment and differential diagnosis of pulmonary lesions [52].

## 11. Interventional Procedures and Pulmonary CEUS

CEUS has also taken on an increasingly important role in guiding percutaneous biopsies, owing to its ability to more precisely identify viable and vascularized areas within lesions, thereby avoiding necrotic zones and improving diagnostic accuracy compared with conventional ultrasound. Although evidence in the pulmonary field remains limited, several studies have demonstrated the effectiveness of CEUS in ultrasound-guided biopsy of peripheral subpleural lung lesions, where it enables precise needle targeting of the most representative tumor area. It is advantageous in cases where the lesion is associated with atelectasis, as it allows differentiation from collapsed tissue and identification of vascular structures to be avoided during the interventional procedure [1,53,54,55,56,57]

Several studies have highlighted the role of CEUS in biopsy procedures and its advantages in interventional practice.

**Figure 6:**
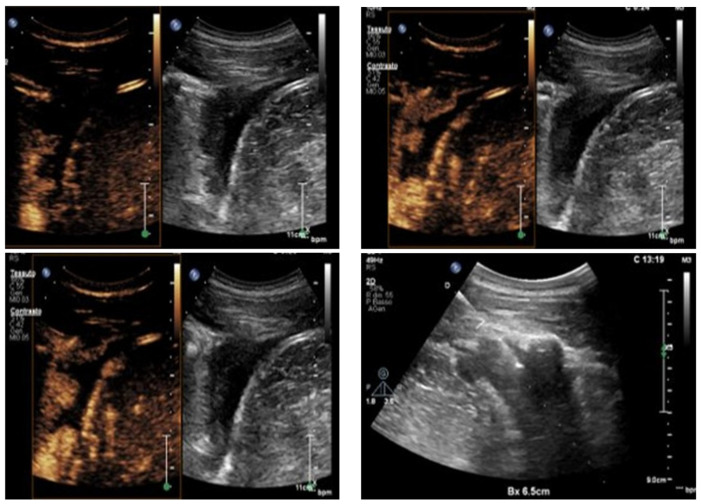
Contrast-enhanced ultrasound (CEUS) findings of multiple pleuro-pulmonary solid nodules with histologically confirmed lung adenocarcinoma. Contrast-enhanced ultrasound demonstrates multiple solid pleuro-pulmonary nodules showing intralesional vascularization. The final diagnosis of lung adenocarcinoma was established by ultrasound-guided biopsy.

In 2019, a study by Fu et al. showed that CEUS improves the diagnostic yield of biopsy in subpleural lung lesions by allowing the identification of necrosis in 34.5% of cases and modification of the biopsy needle path in 27.6% of cases, particularly for lesions ≥5 cm [58]. The diagnostic accuracy reached 98.3%, with only a few mild complications, thus confirming the safety and effectiveness of CEUS before biopsy [58]. Liang et al. studied 120 patients with thoracic lesions and demonstrated that CEUS allows better identification of necrotic and atelectatic areas (40.7% vs. 16.7%), leading to modification of the needle path in 48.1% of cases. The biopsy success rate was higher in the CEUS group (96.3% vs. 80.3%), and minor complications were fewer (3.7% vs. 18.2%). CEUS is therefore useful for identifying viable tissue, increasing diagnostic accuracy, and reducing complications, particularly in necrotic, hypovascular, or atelectatic lesions [59]. Wang et al. evaluated the use of CEUS in the diagnosis and biopsy guidance of peripheral lung lesions in 33 patients (22 malignant and 11 benign). CEUS did not show significant differences in quantitative parameters between benign and malignant lesions but allowed better identification of necrotic areas compared with conventional ultrasound (51.5% vs. 27.3%), achieving a biopsy accuracy of 96.9% with no major complications. The contrast AT was shorter in acute pneumonia lesions, suggesting a potential differential diagnostic value for this technique. CEUS improves target selection for biopsy and can assist in differentiating acute inflammation from other pulmonary lesions, representing a useful complementary technique for the diagnosis of peripheral lung lesions [60]. In a 2021 prospective study, the authors evaluated CEUS-guided pleural biopsy in 460 patients with pleural lesions, demonstrating high diagnostic accuracy (98.9%) and a significant improvement in yield compared with conventional ultrasound owing to better identification of necrosis and peripheral vessels. The microbiological yield in infectious lesions was 71.9%, and no serious adverse events were reported in this study. CEUS thus confirms itself as a safe, minimally invasive, and highly effective technique for the diagnosis of both malignant and infectious pleural [61]. Quarato et al., in a 2021 prospective randomized study, compared CEUS-guided transthoracic percutaneous biopsy with conventional biopsy in 232 patients with subpleural lesions. They found that CEUS allowed better identification of necrotic areas as the lesion size increased (from 8% to 31%). However, CEUS did not show a significant advantage in diagnostic accuracy (94.0% vs. 89.7%; *p* = 0.34) for lesions measuring 1–2 cm or 2–5 cm. Both techniques demonstrated excellent diagnostic performance (AUC ≥ 0.80) and a high safety profile, with only minimal and self-limiting complications [62]. In a further retrospective study conducted on 92 patients, the use of CEUS allowed modification of the biopsy needle path in 43.5% of cases by identifying viable areas within subpleural pulmonary lesions and distinguishing them from necrotic or hypoperfused regions that were not visible with conventional ultrasound. The rate of satisfactory samples was 100%, with a diagnostic accuracy of 97.8% and no major complications observed. CEUS performed prior to biopsy improves target selection, optimizes diagnostic yield, and reduces procedural risks [57]. Zhou et al. compared ultrasound-guided percutaneous lung biopsy with and without contrast enhancement in 345 patients with subpleural lesions. In malignant cases, CEUS provided a significantly higher number and percentage of tumor cells, with a high-quality cellularity (HQC) rate of 86.3% compared to 75.3% with conventional ultrasound, showing the greatest improvement in the biopsies of larger lesions. CEUS increased both cellular yield and tissue quality, confirming it as a more effective technique for obtaining adequate samples in peripheral pulmonary [56] (Figure 6). Ye et al. retrospectively compared the effectiveness of high-frequency contrast-enhanced ultrasound (HF-CEUS)-guided pleural biopsy with that of traditional high-frequency ultrasound (HFU) in 144 patients. HF-CEUS showed a higher success rate (93.2% vs. 81.4%) and fewer complications (2.7% vs. 12.9%) owing to the improved identification of necrotic areas, lesion margins, and large vessels. The authors concluded that HF-CEUS enhances the safety and efficacy of pleural biopsy, enabling more precise sampling and reducing the risk of complications compared with conventional ultrasound [63]. Furthermore, the role of CEUS combined with rapid on-site evaluation (ROSE) has been assessed in the diagnosis of peripheral lung lesions. In a cohort of 80 patients divided into two groups, CEUS achieved a success rate of 97.6% compared to 84% with conventional ultrasound, with no significant complications reported. It also improved the differentiation between benign lesions (showing homogeneous enhancement) and malignant lesions (showing heterogeneous enhancement). The ΔAT parameter (difference between the contrast arrival times in the lesion and pulmonary parenchyma) was a reliable indicator of malignancy, with a threshold value of 2.05 s (AUC = 0.914). CEUS combined with ROSE thus increases the diagnostic accuracy and safety of ultrasound-guided lung biopsy while providing valuable information for distinguishing between benign and malignant lesions [64]. Wang et al., in a multicenter retrospective study, compared the effectiveness of ultrasound-guided transthoracic needle biopsy (PTNB) with and without CEUS in 1027 patients with peripheral lung lesions. CEUS resulted in greater tissue adequacy (98.2% vs. 95.7%) and diagnostic accuracy (96.9% vs. 94.2%), with a significant advantage, particularly in lesions measuring 2–7 cm, but not in those larger than 7 cm. Moreover, CEUS reduced false negatives and improved visualization of necrotic areas and lesion margins, thereby confirming its role in optimizing the diagnostic yield of medium-sized lesions [65]. In a further study, CEUS-guided percutaneous biopsy performed on 237 patients with peripheral lung lesions confirmed high diagnostic accuracy (95.8% vs. 89.9%) and required fewer needle passes (2.58 vs. 2.90), with a significant advantage for lesions ≥ 5 cm, owing to improved identification of viable areas compared with conventional ultrasound-guided biopsy. Overall, CEUS proved to be a more efficient, safe, and precise technique, particularly useful in the management of larger pulmonary lesions [66]. In a 2025 study, the authors compared CEUS and contrast-enhanced CT (CECT) for the biopsy of peripheral lung lesions in 420 patients. CEUS demonstrated greater procedural efficiency, requiring fewer pleural punctures (2.5 vs. 4.1) and shorter procedure times (24 vs. 42 min), with a lower incidence of pneumothorax (3.1% vs. 8%). Diagnostic accuracy was higher for medium-sized lesions (3–6 cm), whereas CECT performed better for smaller lesions (<3 cm). For masses larger than 6 cm, the two techniques showed comparable results. In summary, CEUS represents a faster and safer technique for medium-sized lesions, whereas CECT remains preferable for smaller ones [67]. Sun et al. recently conducted a meta-analysis of 16 studies including 3459 patients, comparing CEUS-guided biopsy with conventional ultrasound (US)-guided biopsy for thoracic and pulmonary lesions. CEUS demonstrated a success rate of 99.2%, diagnostic accuracy of 96%, and fewer complications than US. By allowing the precise identification of viable and vascularized areas while avoiding necrotic regions, CEUS improves both the diagnostic yield and procedural safety. The authors concluded that CEUS represents the preferred technique for biopsy of thoracic and peripheral pulmonary lesions [68].

In conclusion, performing CEUS before percutaneous biopsy allows evaluation of tissue viability and perfusion, selection of optimal sampling sites, and reduction of complication risk, making it a valuable supportive technique for interventional planning.

## 12. Discussion

CEUS represents a versatile imaging modality for the evaluation of pleuropulmonary diseases owing to its ability to dynamically assess tissue perfusion in real time. When combined with the morphological information provided by conventional B-mode ultrasound, it enables a bedside evaluation of both structural and microvascular characteristics of peripheral lung lesions. Evidence from recent studies suggests that CEUS may improve the characterization of subpleural lesions and contribute to the differentiation between benign and malignant processes, as well as inflammatory and ischemic conditions. However, these findings should be interpreted with caution, as many available studies are retrospective, single-center investigations performed in selected patient populations and lesions accessible to ultrasound examination.

In the differential diagnosis of consolidative syndromes, CEUS can provide useful functional information by identifying microvascular perfusion patterns that reflect the underlying pathophysiology of the disease. For example, early and relatively homogeneous enhancement is commonly associated with inflammatory or infectious lesions, whereas delayed and heterogeneous enhancement with early washout may suggest a neoplastic or ischemic etiology. Nevertheless, these patterns are not entirely specific and may overlap with other conditions such as organizing pneumonia, granulomatous diseases, or necrotic tumors, highlighting the need for careful interpretation within the broader clinical and radiological context.

From a practical perspective, CEUS may support clinical decision-making in several settings. In pneumonia and pleural infections, it can help identify non-perfused or necrotic areas within consolidations, potentially facilitating drainage procedures and monitoring treatment response. In suspected pulmonary embolism, the detection of avascular peripheral consolidations may support the diagnosis of pulmonary infarction, particularly in selected cases where CT pulmonary angiography is inconclusive or not immediately available. Similarly, in the context of viral pneumonia, including COVID-19, CEUS may provide additional information on perfusion heterogeneity and possible microvascular alterations during follow-up.

In patients with peripheral lung neoplasms, CEUS may contribute to the evaluation of tumor vascularization and angiogenic activity, helping to differentiate between pulmonary and bronchial arterial supply and to identify viable areas for biopsy. Quantitative perfusion parameters, such as enhancement time and washout kinetics, have shown promising correlations with histopathological findings in some studies and may support the development of multiparametric diagnostic models. However, these results require further validation in larger prospective studies with standardized CEUS protocols.

Overall, CEUS should be considered a complementary imaging modality that provides real-time functional information on pulmonary microvascular perfusion (Table 3). While it can enhance the evaluation of peripheral lung lesions and assist in interventional planning, it does not replace established cross-sectional imaging techniques such as contrast-enhanced CT, CT pulmonary angiography, or PET/CT, which remain the reference standards for comprehensive thoracic assessment and disease staging.

## 13. CEUS Limitations

Despite the promising results reported in the literature, several limitations of the available evidence should be considered. Many of the studies investigating the role of CEUS in pleuropulmonary diseases are retrospective and conducted in single-center settings, often with relatively limited sample sizes. These factors may introduce selection bias and limit the generalizability of the reported diagnostic performance. In addition, there is considerable heterogeneity among studies in terms of patient populations, lesion types, CEUS acquisition protocols, and the perfusion parameters analyzed. Differences in the definition and interpretation of variables such as arrival time, washout time, and enhancement patterns may further contribute to variability across studies [19,20]. Consequently, although the overall evidence supports the clinical usefulness of CEUS in the evaluation of peripheral pulmonary lesions, the results should be interpreted with caution. Future prospective multicenter studies with standardized imaging protocols and quantitative perfusion analysis are needed to further validate the diagnostic role of thoracic CEUS and improve the reproducibility of the technique.

Another relevant aspect to consider is the operator-dependent nature of thoracic ultrasound and CEUS. Recent efforts have focused on developing structured and competency-based training programs for thoracic ultrasound, aimed at improving standardization and reproducibility in clinical practice [69]. In this context, the integration of CEUS into thoracic imaging raises the question of whether specific training pathways may be required beyond conventional ultrasound skills. While CEUS can be considered a natural extension of thoracic ultrasound, its correct application requires familiarity with contrast-specific imaging modes, appropriate contrast agent administration, and the interpretation of dynamic perfusion patterns [70]. These additional competencies suggest that dedicated training modules may be beneficial, particularly in centers performing advanced thoracic ultrasound or interventional procedures. Future educational initiatives and standardized training frameworks may help improve the consistency and clinical implementation of thoracic CEUS [71].

## 14. Future Perspectives

Artificial intelligence and machine learning algorithms are also emerging as promising tools for ultrasound image analysis [72,73]. Recent studies have explored automated detection of lung ultrasound patterns, including B-lines, pleural irregularities, and subpleural consolidations, demonstrating encouraging results in terms of diagnostic accuracy and interobserver reproducibility [72,74,75]. These technologies may help reduce operator dependency, which remains one of the main limitations of thoracic ultrasound techniques. In addition, the application of artificial intelligence to CEUS datasets could allow automated assessment of perfusion dynamics and quantitative characterization of microvascular patterns, potentially supporting the differentiation between inflammatory, ischemic, and neoplastic lesions [76]. Early experiences in cardiovascular and pulmonary imaging suggest that AI-assisted ultrasound analysis may facilitate clinical decision-making and improve workflow efficiency [45,77,78]. The integration of quantitative CEUS parameters with clinical data and other imaging modalities may also contribute to the development of multiparametric diagnostic models and decision-support systems, potentially improving the stratification of pulmonary lesions and guiding personalized therapeutic strategies [76]. Although these approaches remain largely investigational, they represent an important step toward a more standardized, reproducible, and data-driven use of thoracic ultrasound, expanding its potential role in precision medicine and real-time bedside imaging.

## 15. Conclusions

CEUS is a safe, noninvasive, and highly informative technique for the evaluation of pleuropulmonary diseases. By integrating morphological and perfusion data, diagnostic precision is enhanced, personalized clinical management is supported, and conventional imaging techniques are complemented, paving the way for broader implementation in clinical practice and future research in pulmonary microvascular imaging.

However, while CEUS provides valuable real-time information on microvascular perfusion and may improve the characterization of peripheral lung lesions, its role should be viewed as complementary to conventional imaging modalities rather than as a replacement for cross-sectional imaging techniques.

## Figures and Tables

**Table 1:** Quantitative contrast-enhanced ultrasound (CEUS) perfusion parameters reported in the literature for the characterization of subpleural pulmonary lesions. The table summarizes the main quantitative CEUS parameters investigated in published studies, including reported cut-off values where available. These parameters provide functional information on lesion vascularization but show variable reproducibility across studies, reflecting differences in study design, patient populations, and CEUS acquisition protocols. Abbreviations: CEUS, contrast-enhanced ultrasound; AT, arrival time; TDOA, time difference of arrival; ΔAT, difference in arrival time; WT, washout time; PI, peak intensity; TTP, time to peak.

CEUS Parameter	Reported Cut-Off/Typical Values	Interpretation	Consistency	Main Limitations
Arrival Time (AT)	Pulmonary arterial supply: ≤5–7 s Bronchial arterial supply: >6–7 s	Delayed enhancement suggests bronchial systemic vascularization typical of malignant lesions	Moderate	Significant overlap between inflammatory and malignant lesions
Time Difference of Arrival (TDOA)	Cut-off ≈ 2.4 s	Higher values associated with malignant lesions	Moderate–good	Limited external validation
Washout Time (WT)	<60 s early washout	Strongly associated with malignancy	Relatively consistent	High specificity but low sensitivity
Peak Intensity (PI)	Higher PI in malignant lesions in some studies	Reflects increased neoangiogenesis	Variable	Lack of standardized thresholds
Time To Peak (TTP)	Shorter TTP in malignant pleural disease in some cohorts	Rapid contrast accumulation due to abnormal vascularization	Variable	Considerable inter-study variability
ΔAT (Difference Between Lesion And Lung)	≥2.0–2.5 s suggestive of malignancy	Reflects delayed systemic perfusion compared with pulmonary circulation	Moderate	Requires precise timing measurement
Non-Enhancing Necrotic Areas	Frequently present in malignant lesions; irregular margins	Necrosis due to tumoral ischemia	Moderate	Also observed in abscesses and infarctions

**Table 2:** Summary of key studies evaluating contrast-enhanced ultrasound (CEUS) parameters for the differential diagnosis of benign and malignant subpleural pulmonary lesions. The table summarizes the main clinical studies investigating CEUS in subpleural lung lesions, including sample size, evaluated perfusion parameters, and reported diagnostic performance. Abbreviations: CEUS, contrast-enhanced ultrasound; AT, arrival time; TDOA, time difference of arrival; TTP, time to peak; TIC, time–intensity curve; AUC, area under the curve; SPLs, subpleural pulmonary lesions.

Study	Sample Size	Lesion Type	Key CEUS Parameters	Main Findings	Diagnostic Performance
Tang et al. (2020) [13]	96	Peripheral pulmonary lesions	TDOA	TDOA significantly higher in malignant lesions	AUC 0.894; Sensitivity 86.3%; Specificity 88.9%
Bi et al. (2021) [14]	812	Subpleural pulmonary lesions	AT difference, vascular sign, non-enhancing areas	Multiparametric CEUS + B-mode model for malignancy prediction	C-statistic 0.974–0.980
Findeisen et al. (2022) [16]	63	Pleural lesions	Enhancement intensity, homogeneity	Marked enhancement associated with malignancy	Sensitivity 78.6%; Specificity 74.1%
Yang et al. (2022) [17]	50	Pleural thickening	AT, TTP, TIC parameters	Malignant lesions showed faster and heterogeneous enhancement	AUC 0.975 (combined model)
Quarato et al. (2023) [18]	317	Peripheral consolidations	AT, washout time	Limited diagnostic value of isolated CEUS parameters	Accuracy 47–53%

**Table 3:** Proposed positioning of CEUS within the diagnostic pathway of pleuropulmonary diseases. The table summarizes clinical scenarios in which CEUS may be considered recommended, optional, or investigational based on currently available evidence and guideline recommendations. CEUS should generally be interpreted as a complementary imaging modality integrated with conventional ultrasound and standard cross-sectional imaging techniques. Abbreviations: CEUS, contrast-enhanced ultrasound; CT, computed tomography; PET/CT, positron emission tomography/computed tomography; CTPA, CT pulmonary angiography.

Clinical Scenario	Role of CEUS	Clinical Purpose	Reference Imaging Standard	Level of Evidence
Peripheral subpleural pulmonary lesions visible on ultrasound	Recommended (adjunctive)	Characterization of perfusion patterns, identification of necrotic areas, biopsy targeting	Contrast-enhanced CT	Moderate
Ultrasound-guided biopsy of pleural or subpleural lesions	Recommended	Identification of viable tissue and avoidance of necrotic areas	CT or PET/CT for staging	Good
Pneumonia with suspected necrosis or abscess	Optional	Identification of non-perfused areas and monitoring of treatment response	Chest CT when clinically indicated	Moderate
Complex pleural effusion or empyema	Optional	Evaluation of pleural vascularization and septations; guidance for drainage	CT in complicated cases	Moderate
Suspected peripheral pulmonary infarction	Optional	Detection of avascular wedge-shaped consolidations	CT pulmonary angiography (CTPA)	Limited–moderate
Follow-up of peripheral pulmonary lesions during therapy	Optional	Assessment of perfusion changes and lesion viability	CT or PET/CT depending on pathology	Limited
Deep parenchymal lung lesions not in contact with pleura	Not recommended	Limited acoustic window	Contrast-enhanced CT	Strong
Staging of lung cancer	Investigational/not recommended	Limited field of view	PET/CT, contrast-enhanced CT	Strong
Primary diagnosis of pulmonary embolism	Investigational	May support diagnosis in selected cases	CT pulmonary angiography	Strong

## Data Availability

No new data were created or analyzed in this study.
